# Supplementary material for: Biodiversity mediates ecosystem sensitivity to climate variability
Source: Commun Biol. 2022 Jun 27;5:628. doi: 10.1038/s42003-022-03573-9 (PMC9237054; doi:10.1038/s42003-022-03573-9)
Supplement: Supplementary file 2 — Supplementary Information [file 42003_2022_3573_MOESM2_ESM.pdf]

Supplementary material for:

Biodiversity mediates ecosystem sensitivity to climate variability

Brunno F. Oliveira [1,2*]

Frances C. Moore [1]

Xiaoli Dong [1]

Affiliations:

[1] Department of Environmental Science and Policy, University of California – Davis, Davis, CA 95616

[2] Current address: Centre for the Synthesis and Analysis of Biodiversity (CESAB) – Foundation for Biodiversity Research (FRB), Montpellier, France

* Corresponding author: brunno.oliveira@me.com (ORCID: 0000-0002-2982-149X)

Supplementary Text

Validation of geographical patterns in biodiversity

We validated estimated species richness patterns used in our study (which was generated by overlaying species range maps) with species richness collected at the plot-level. Here, we used plot-data extracted from the vegetation inventory database SALVIAS (Synthesis and Analysis of Vegetation Inventories Across Scales). The scale of our analyses (~50 km^2 grid-cells at the equator) means that we can infer regional-scale biodiversity patterns. However, as field data are often collected at a much smaller scale (i.e., a few square meters), these data could underestimate landscape- or regional-scale biodiversity patterns. To control for this issue, we aggregated species lists from SALVIAS plots to the grid-cell scale, and calculated species richness across all plots falling within a grid-cell. Moreover, the number of plots at each grid-cell vary widely (minimum = 1, maximum = 44, SD = 5.93), which can potentially result in underestimated regional-scale species richness. We control for this issue by selected only those grid-cells contained at least 4 plots. This resulted in 146 grid-cells with estimated richness from plots (Supplementary Fig. 7). We found a significant and positive correlation between species richness estimated from range maps and that richness estimated from aggregated local-scale plots (Coefficient = 0.87, p-value < 0.001, R^2 = 0.32).

Supplementary Table 1. List of traits used for calculating functional diversity. Completeness (percent of species with at least one recorded trait measurement) varied widely across the 23 traits selected. All traits were used for imputation (see Methods). Traits in bold were selected estimating for functional diversity. N sps., number of species. N obs., number of trait measurements. Completeness (%), percent of species with at least one trait measurement. BIEN, whether traits were extracted from BIEN (1) or not (0). TRY, whether traits were extracted from TRY (1) or not (0).

Trait	N sps.	N obs.	Completeness (%)	BIEN	TRY
C	2884	32440	5.01	1	1
CN	2375	23997	4.12	0	1
**Height**	**7968**	**122807**	**13.83**	**1**	**1**
K	1053	5982	1.83	0	1
**LA**	**6169**	**103045**	**10.71**	**1**	**1**
LADM	5934	179121	10.3	0	1
**LDM**	**3569**	**81809**	**6.2**	**1**	**1**
LDMC	2790	126618	4.84	0	1
LFM	1119	30379	1.94	1	1
Lon	6391	27311	11.09	1	1
LPho	1849	43266	3.21	1	1
LThick	2999	63111	5.21	1	1
**N**	**5015**	**74380**	**8.71**	**1**	**1**
NP	1477	8309	2.56	0	1
**P**	**3062**	**28375**	**5.32**	**1**	**1**
RootDepth	2240	4696	3.89	0	1
SeedBankLon	909	29468	1.58	0	1
SeedGermRate	3871	17837	6.72	0	1
**SeedMass**	**7848**	**122704**	**13.62**	**1**	**1**
**SLA**	**2828**	**30450**	**4.91**	**1**	**0**
StomConduc	1387	32797	2.41	0	1
**WoodDens**	**3444**	**40763**	**5.98**	**1**	**1**

* C, leaf carbon content per leaf dry mass. CN, leaf carbon/nitrogen ratio. Height, maximum plant height. K, leaf potassium content per leaf dry mass. LA, leaf area. LADM, leaf area per leaf dry mass. LDM, leaf dry mass. LDMC, leaf dry matter content. LFM, leaf fresh mass. Lon, plant lifespan. LPho, leaf photosynthesis rate per leaf area. LThick, leaf thickness. N, leaf nitrogen content per leaf dry mass. NP, leaf nitrogen/phosphorus ratio. P, leaf phosphorus content per leaf dry mass. RootDepth, root rooting depth. SeedBankLon, Seed (seedbank) longevity. SeedGermRate, Seed germination rate. SeedMass, Seed dry mass. SLA, leaf area per leaf dry mass. StomConduc, Stomata conductance per leaf area. WoodDens, wood density (stem dry mass per stem fresh volume).

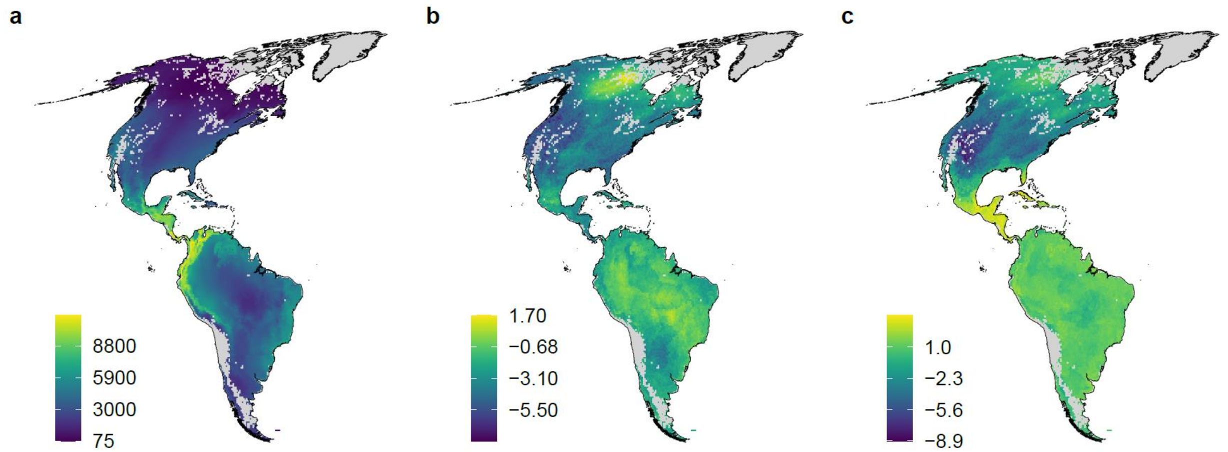

Supplementary Fig. 1 | Geographic distribution of plant biodiversity dimensions across the Western Hemisphere. Patterns of plant species richness (**a**), phylogenetic diversity (**b**) and functional diversity (**c**). Phylogenic and functional diversity are shown in units of standardized effect sizes (see Methods). In (**b**) and (**c**), positive values represent higher phylogenetic or functional diversity than expected by chance given species richness in a grid-cell, whereas negative values represent lower phylogenetic or functional diversity than expected by chance.

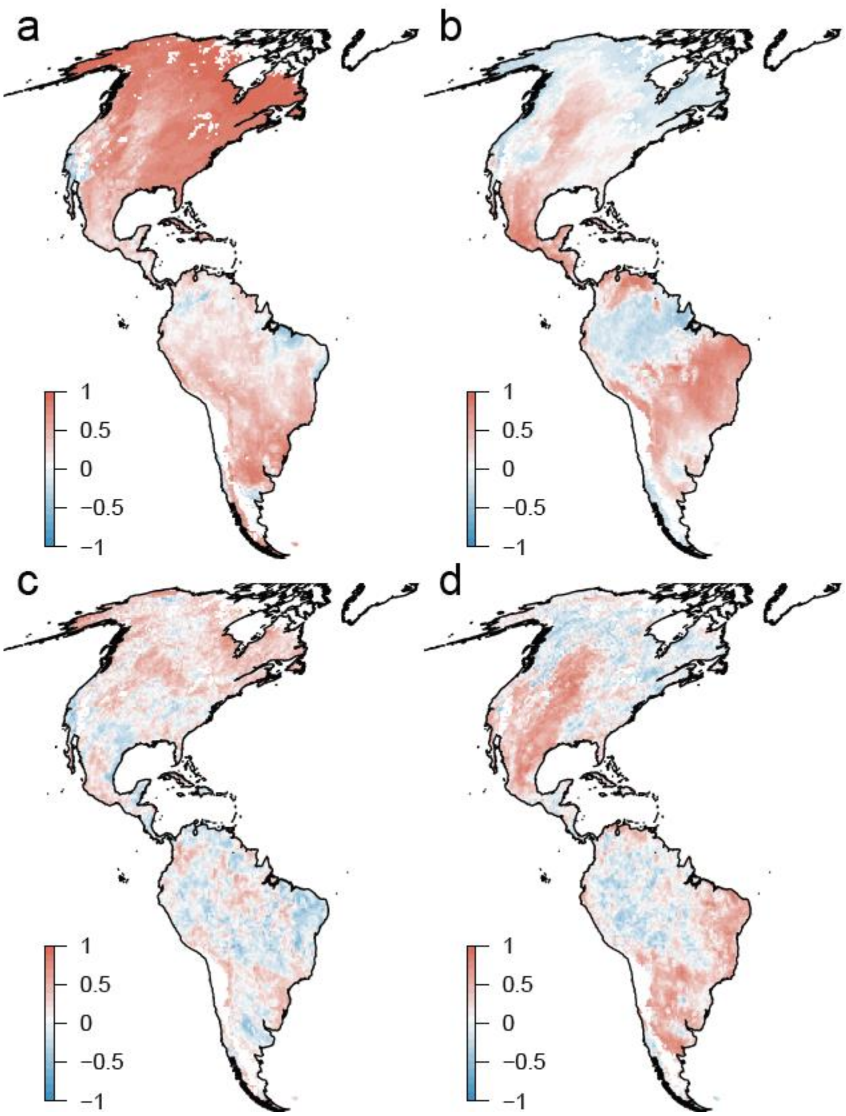

Supplementary Fig. 2 | Geographic patterns in ecosystem sensitivity to temporal variability in temperature and precipitation. Ecosystem sensitivity to seasonality in temperature (**a**) and precipitation (**b**), and to interannual changes in temperature (**c**) and precipitation (**d**). Ecosystem stability represents areas of constant and largely stable productivity conditions despite large climate variability (see Methods). White areas represent extremely sparse or nonexistent vegetation cover and were eliminated from our analyses to reduce the potential impact of noisy data at low EVI values.

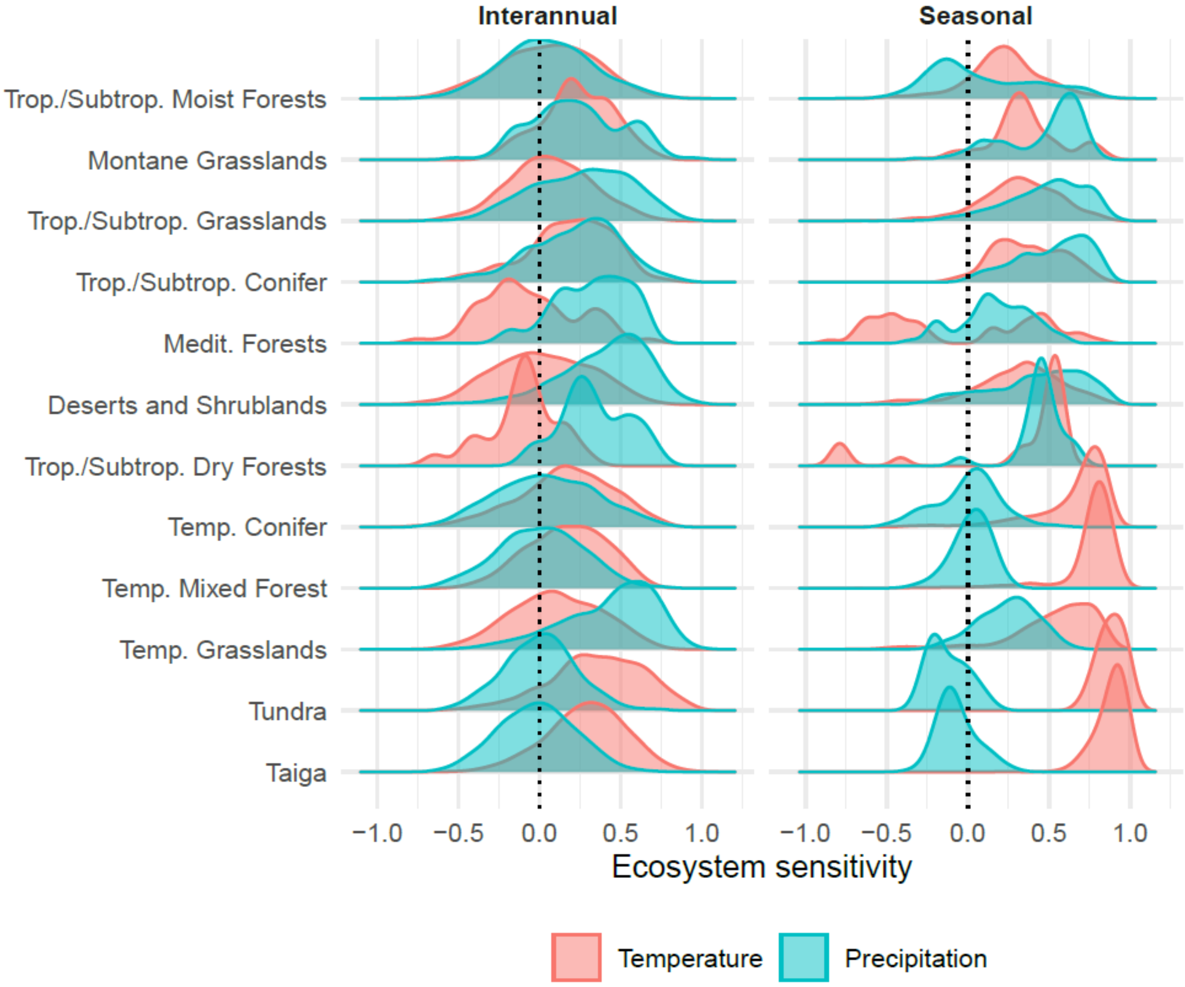

Supplementary Fig. 3 | Variability in ecosystem sensitivity within biomes. Ecosystem sensitivity within biomes varied widely both at the interannual (**a**) and (**b**) temporal scales.

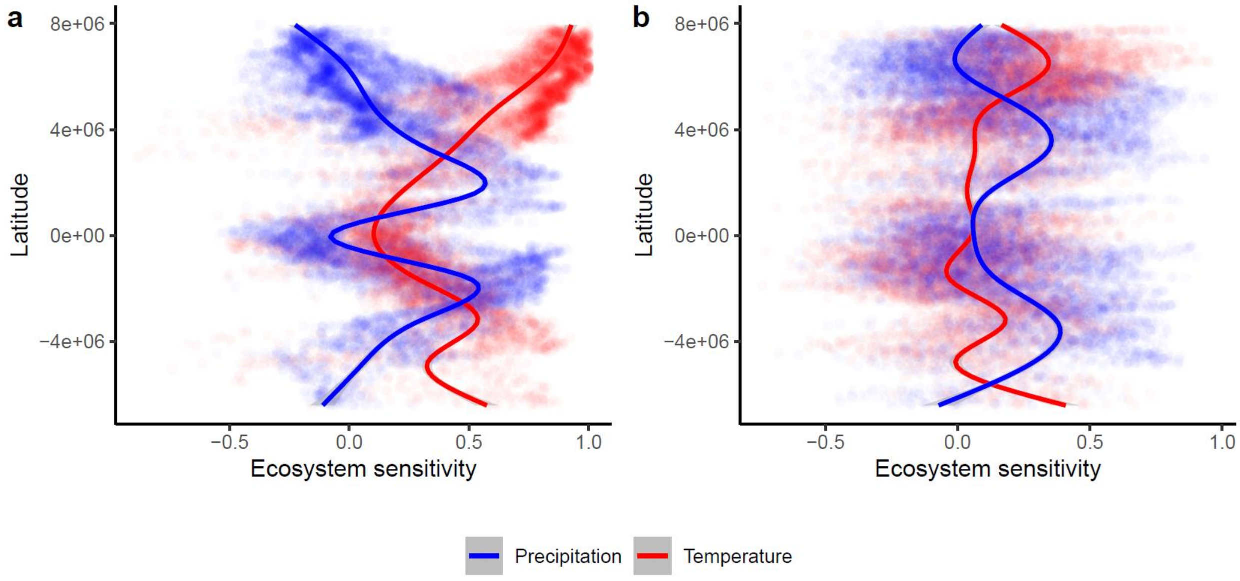

Supplementary Fig. 4 | Latitudinal patterns in ecosystem sensitivity. Latitudinal patterns in ecosystem sensitivity to climate variability at seasonal (**a**) and interannual (**b**) scales. Red and blue lines represent the fit of locally estimated scatterplot smoothing (LOESS) regression for temperature and precipitation, respectively.

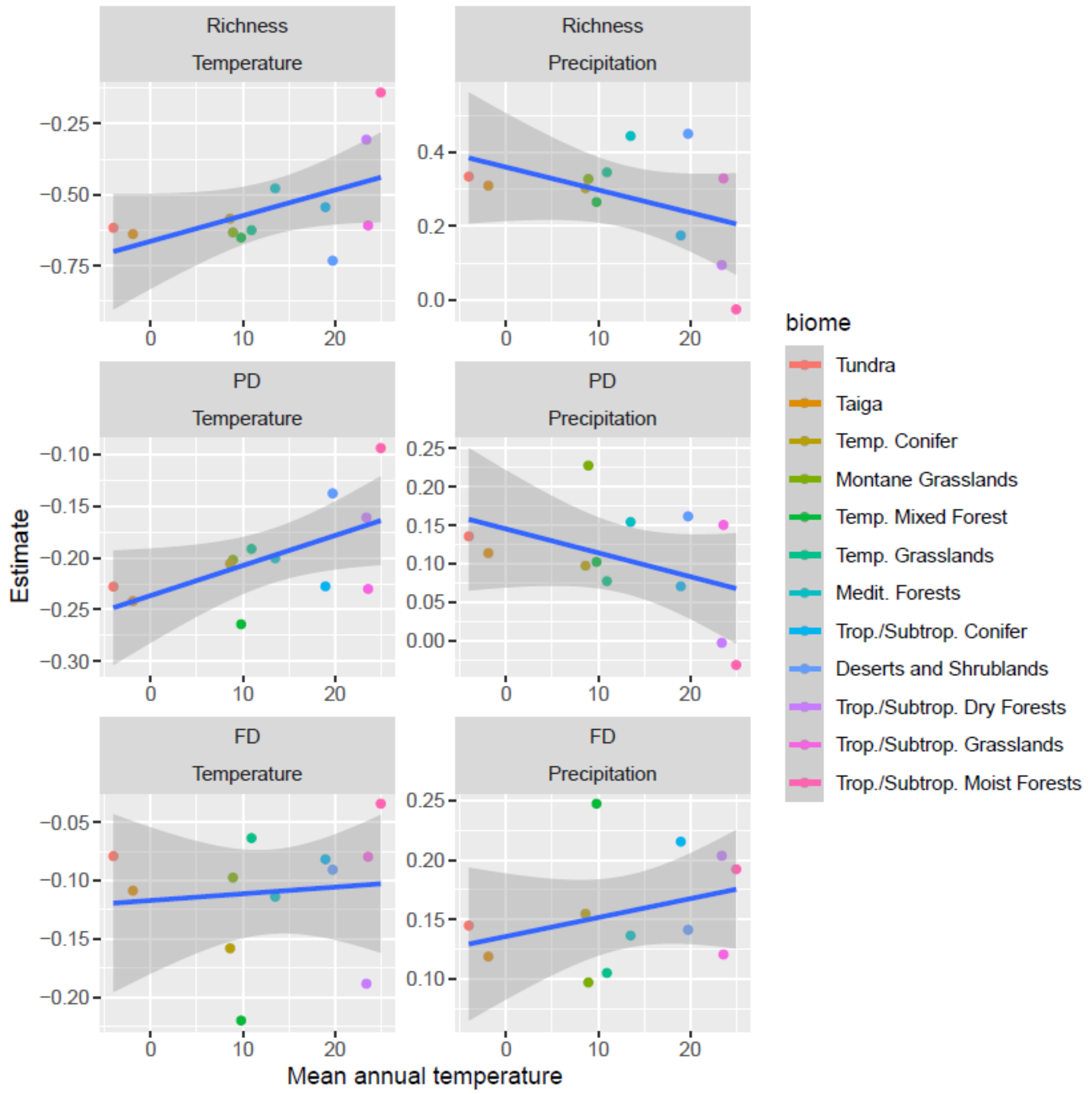

Supplementary Fig. 5 | Biome-level annual mean temperature and biodiversity effects on ecosystem sensitivity at the seasonal-scale. Each panel shows the effect (standardized coefficient from SAR models) of each biodiversity dimension (rows) on ecosystem sensitivity to temperature (left side column) and precipitation (right side column). The x-axes represent mean annual temperature within a biome. Dots represents the effect of each biodiversity dimension on ecosystem sensitivity at each biome. Dots are color coded by biome.

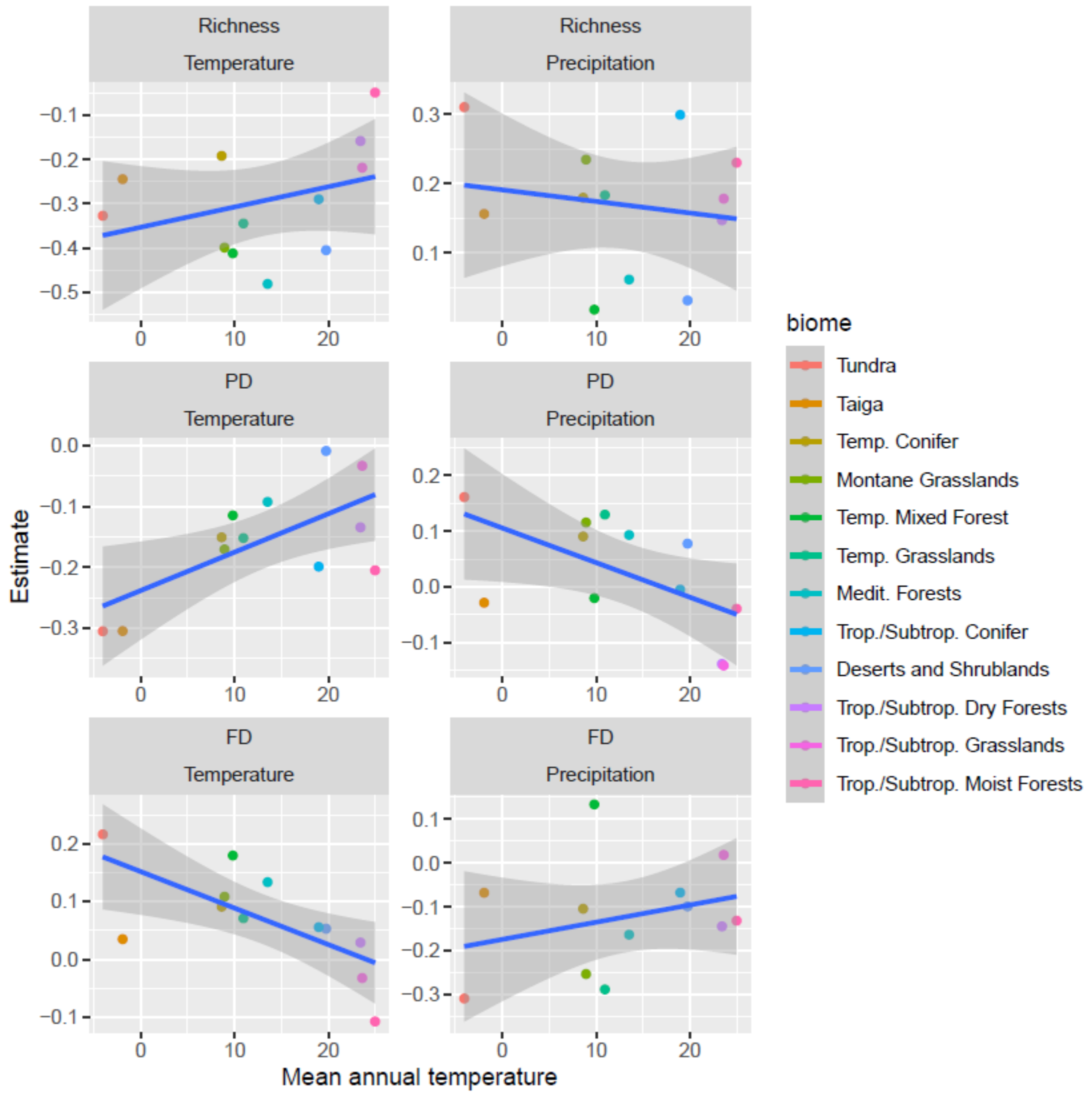

Supplementary Fig. 6 | Biome-level annual mean temperature and biodiversity effects on ecosystem sensitivity at the interannual-scale. Each panel shows the effect (standardized coefficient from SAR models) of each biodiversity dimension (rows) on ecosystem sensitivity to temperature (left side column) and precipitation (right side column). The x-axes represent mean annual temperature within a biome. Dots represents the effect of each biodiversity dimension on ecosystem sensitivity at each biome. Dots are color coded by biome.

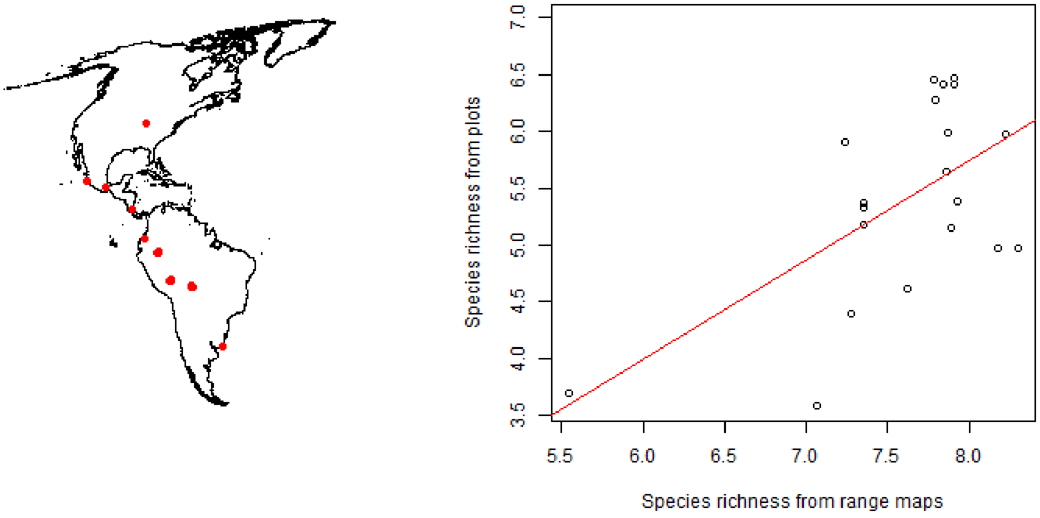

Supplementary Fig. 7 | Validation of grid-cell scale species richness patterns. The map on the right-side panel denotes the distribution of grid-cell scale (~50 km^2) estimated species richness values from aggregated local-scale plots. The scatterplot on the left-side panel shows there is a positive correlation between species richness estimated from range maps and that richness estimated from aggregated local-scale plots. Species lists from plots were extracted from the vegetation inventory database SALVIAS. Species richness values are log-scaled.

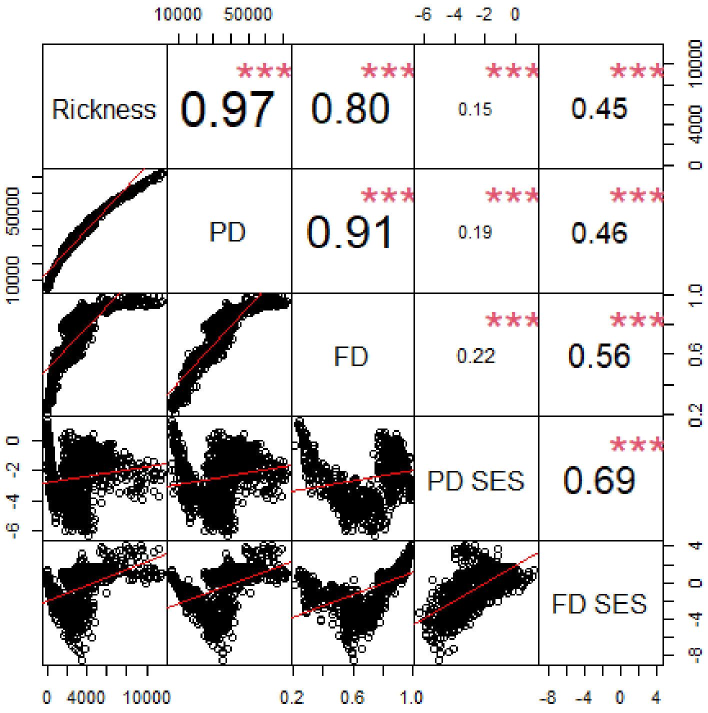

Supplementary Fig. 8 | Pairwise correlation among biodiversity dimensions. On top the values of Pearson's correlation coefficients, plus the significance of these correlations. On bottom, bivariate scatterplots, with a fitted line in red from a linear model. Species richness correlates strongly with raw PD and FD than with the SES version of PD and FD. ***, P < 0.001.
