# Supplementary material for: Biodiversity mediates ecosystem sensitivity to climate variability
Source: Commun Biol. 2022 Jun 27;5:628. doi: 10.1038/s42003-022-03573-9 (PMC9237054; doi:10.1038/s42003-022-03573-9)
Supplement: Supplementary file 3 — Reporting summary [file 42003_2022_3573_MOESM3_ESM.pdf]

nature portfolio

Corresponding author(s): Brunno Freire Oliveira

Last updated by author(s): Jun 6, 2022

Reporting Summary

Nature Portfolio wishes to improve the reproducibility of the work that we publish. This form provides structure for consistency and transparency in reporting. For further information on Nature Portfolio policies, see our Editorial Policies and the Editorial Policy Checklist.

Statistics

For all statistical analyses, confirm that the following items are present in the figure legend, table legend, main text, or Methods section.

n/a	Confirmed	
☐	☒	The exact sample size (*n*) for each experimental group/condition, given as a discrete number and unit of measurement
☐	☒	A statement on whether measurements were taken from distinct samples or whether the same sample was measured repeatedly
☐	☒	The statistical test(s) used AND whether they are one- or two-sided<br>*Only common tests should be described solely by name; describe more complex techniques in the Methods section.*
☐	☒	A description of all covariates tested
☐	☒	A description of any assumptions or corrections, such as tests of normality and adjustment for multiple comparisons
☐	☒	A full description of the statistical parameters including central tendency (e.g. means) or other basic estimates (e.g. regression coefficient) AND variation (e.g. standard deviation) or associated estimates of uncertainty (e.g. confidence intervals)
☐	☒	For null hypothesis testing, the test statistic (e.g. *F*, *t*, *r*) with confidence intervals, effect sizes, degrees of freedom and *P* value noted<br>*Give P values as exact values whenever suitable.*
☒	☐	For Bayesian analysis, information on the choice of priors and Markov chain Monte Carlo settings
☒	☐	For hierarchical and complex designs, identification of the appropriate level for tests and full reporting of outcomes
☐	☒	Estimates of effect sizes (e.g. Cohen's *d*, Pearson's *r*), indicating how they were calculated

Our web collection on statistics for biologists contains articles on many of the points above.

Software and code

Policy information about availability of computer code

Data collection	All analyses were performed in R version 4.0.2.<br>MODIS EVI data was downloaded using the R package MODIS version 1.2.2.<br>Climate variables were downloaded from http://www.climatologylab.org/terraclimate.html<br>Data on plants distribution and phylogenies were downloaded using the R package BIEN version 1.2.4.<br>Data on plant traits were downloaded from BIEN (using the R package BIEN version 1.2.4) and from TRY (retrieved on 5/28/2021 from web portal https://www.try-db.org).
Data analysis	All analyses were performed in R version 4.0.2.<br>Spatial simultaneous autoregressive (SAR) models were fitted using the R package spatialreg 1.1-5.

For manuscripts utilizing custom algorithms or software that are central to the research but not yet described in published literature, software must be made available to editors and reviewers. We strongly encourage code deposition in a community repository (e.g. GitHub). See the Nature Portfolio guidelines for submitting code & software for further information.

Data

Policy information about availability of data

All manuscripts must include a data availability statement. This statement should provide the following information, where applicable:

- Accession codes, unique identifiers, or web links for publicly available datasets
- A description of any restrictions on data availability
- For clinical datasets or third party data, please ensure that the statement adheres to our policy

This work was based entirely on data that are publicly available through National Aeronautics and Space Administration (NASA; https://modis.gsfc.nasa.gov/data/

dataprod/mod13.php), Terraclimate (http://www.climatologylab.org/terraclimate.html), Terrestrial Ecoregions of the World (https:// www.worldwildlife.org/), BIEN (http://bien.nceas.ucsb.edu/bien/) and TRY (https://www.try-db.org).

Field-specific reporting

Please select the one below that is the best fit for your research. If you are not sure, read the appropriate sections before making your selection.

☐ Life sciences ☐ Behavioural & social sciences ☒ Ecological, evolutionary & environmental sciences

For a reference copy of the document with all sections, see nature.com/documents/nr-reporting-summary-flat.pdf

Ecological, evolutionary & environmental sciences study design

All studies must disclose on these points even when the disclosure is negative.

Study description	To test the biogeographical coupling between large-scale patterns of plant diversity and the sensitivity vegetation productivity to climate variability
Research sample	Vascular plants distributed across the Western Hemisphere (North, Central and South America)
Sampling strategy	57,500 vascular plant species distributed across the Western Hemisphere (North, Central and South America) at a 0.5° grid resolution (~50 km2 at the equator). Plant data was retrieved from the two most comprehensive assessors on plant distributions, traits and phylogenies (BIEN and TRY) to date. Total number of grid cells used in analyses was 11,527.
Data collection	Plant data was retrieved from the BIEN R package and from the TRY web portal (https://www.try-db.org) retrieved 5/28/2021.<br>Remote sensing vegetation production data (MODIS EVI) was downloaded using the R package MODIS version 1.2.2.<br>Climate data on temperature and precipitation were downloaded from http://www.climatologylab.org/terraclimate.html.<br>Both vegetation and climate data were downloaded as monthly raster files ranging from February 2000 to December 2019.
Timing and spatial scale	Both vegetation and climate data were downloaded as monthly raster files ranging from February 2000 to December 2019.<br>All data (plants, vegetation and climate) were at a 0.5° grid resolution. Analyses were restricted to the Western Hemisphere (North, Central and South America) based on constraints on the accuracy of plant diversity data (as per BIEN's data availability).
Data exclusions	Analyses were restricted to the Western Hemisphere (North, Central and South America) based on constraints on the accuracy of plant diversity data (as per BIEN's data availability).
Reproducibility	All code used in this study will be available upon publication.
Randomization	The model structure include as predictor variables: 1) the three biodiversity dimensions, 2) biome, 3) regional limitation factors (water-limited vs energy-limited), 4) interactions between each biodiversity dimension and 4.1) biomes, and interactions between each biodiversity dimension and 4.2) regional limiting factor.
Blinding	n/a

Did the study involve field work? ☐ Yes ☒ No

Reporting for specific materials, systems and methods

We require information from authors about some types of materials, experimental systems and methods used in many studies. Here, indicate whether each material, system or method listed is relevant to your study. If you are not sure if a list item applies to your research, read the appropriate section before selecting a response.

Materials & experimental systems

n/a	Involved in the study
☒	☐ Antibodies
☒	☐ Eukaryotic cell lines
☒	☐ Palaeontology and archaeology
☒	☐ Animals and other organisms
☒	☐ Human research participants
☒	☐ Clinical data
☒	☐ Dual use research of concern

Methods

n/a	Involved in the study
☒	☐ ChIP-seq
☒	☐ Flow cytometry
☒	☐ MRI-based neuroimaging
